# Supplementary material for: Proposal of Lentzea deserti (Okoro et al. 2010) Nouioui et al. 2018 as a later heterotypic synonym of Lentzea atacamensis (Okoro et al. 2010) Nouioui et al. 2018 and an emended description of Lentzea atacamensis
Source: PLoS One. 2021 Feb 4;16(2):e0246533. doi: 10.1371/journal.pone.0246533 (PMC7861442; doi:10.1371/journal.pone.0246533)

**Proposal of *Lentzea deserti* (Okoro *et al*. 2010) Nouioui et al. 2018 as a later heterotypic synonym of *Lentzea atacamensis* (Okoro et al. 2010) Nouioui et al. 2018: emended description of *Lentzea atacamensis***

Mo Ping ^1^, Zhao Yun-Lin ^1*^, Jun Liu^1^, Gao Jian ^3^, Xu Zheng-Gang ^1,2*^

^1^Hunan Research Center of Engineering Technology for Utilization of Environmental and Resources Plant, Central South University of Forestry and Technology, Changsha 410004 Hunan, China.

^2^College of Forestry, Northwest A & F University, Yangling 712100 Shaanxi, China.

^3^School of Life Science, Hunan University of Science and technology, Xiangtan 411201 Hunan, China.

^*^Correspondence

Email: [zyl8291290@163.com](mailto:zyl8291290@163.com) (ZYL); [xuzhenggang@nwafu.edu.cn](mailto:xuzhenggang@nwafu.edu.cn) (XZG).

**S1 Fig.** Cultural characteristics of stains in different medium after 21d of incubation at 28°C.

Note: 1, *L. atacamensis* CGMCC 4.5536^T^ (=C61^T^=DSM 45479^T^); 2, *L. deserti* CGMCC 4.5535^T^ (=C68^T^=DSM 45480^T^); No.1, Gause’s synthetic No. 1 medium.


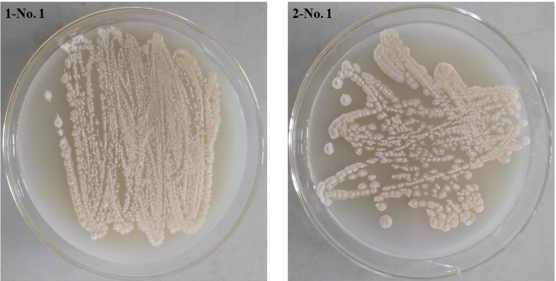


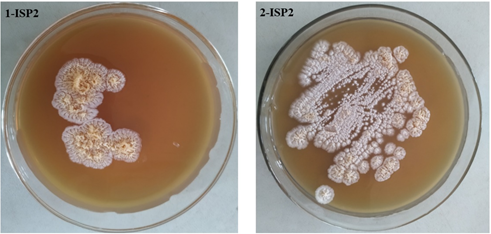


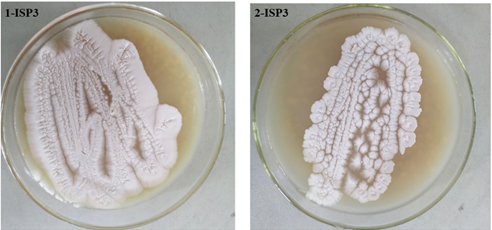


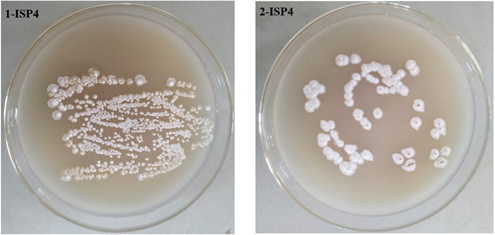


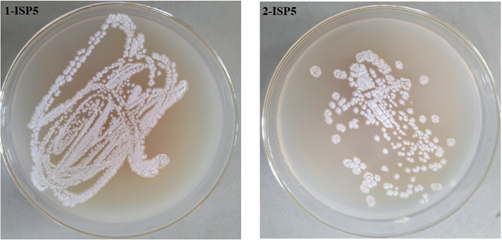


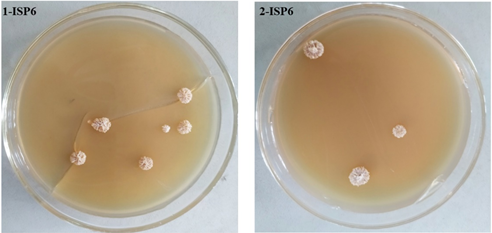


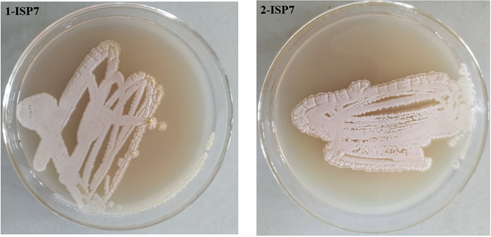

Supplement: S1 Fig — (DOCX) [file pone.0246533.s001.docx]
